# DFT studies on one-electron oxidation and one-electron reduction for 2- and 4-aminopyridines

**DOI:** 10.1007/s00894-012-1446-8

**Published:** 2012-05-15

**Authors:** Ewa D. Raczyńska, Tomasz M. Stępniewski, Katarzyna Kolczyńska

**Affiliations:** 1 Department of Chemistry, Warsaw University of Life Sciences (SGGW), ul. Nowoursynowska 159 c, 02-776 Warsaw, Poland; 2 Interdisciplinary Department of Biotechnology, Warsaw University of Life Sciences (SGGW), ul. Nowoursynowska 166, 02-776 Warsaw, Poland

**Keywords:** Aminopyridines, DFT, N-aza effects, NH and CH tautomers, One-electron oxidation, One-electron reduction, π-Electron delocalization

## Abstract

Quantum-chemical calculations {DFT(B3LYP)/6-311+G(d,p)} were performed for all possible tautomers (aromatic and nonaromatic) of neutral 2- and 4-aminopyridines and their oxidized and reduced forms. One-electron oxidation has no important effect on the tautomeric preference for 2-aminopyridine. The amine tautomer is favored. However, oxidation increases the stability of the imine NH tautomer, and its contribution in the tautomeric mixture cannot be neglected. In the case of 4-aminopyridine, one-electron oxidation increases the stability of both the amine and imine NH tautomers. Consequently, they possess very close energies. As major tautomers, they dictate the composition of the tautomeric mixture. The CH tautomers may be considered as very rare forms for both neutral and oxidized aminopyridines. A reverse situation takes place for the reduced forms of aminopyridines. One-electron reduction favors the C3 atom for the labile proton for both aminopyridines. This may partially explain the origin of the CH tautomers for the anionic states of nucleobases containing the exo NH_2_ group.

## Introduction

Intramolecular proton transfer, called prototropic tautomerism or prototropy, is the simplest process that occurs for natural products (bioamines such as histamine, amino acids such as histidine and arginine, nucleobases such as cytosine, thymine, uracil, adenine, and guanine, porphyrins, etc.) [1–5]. This elementary conversion dictates their structure, acid–base properties, hydrogen bond formation, solvent interactions, and other physicochemical properties. It influences also the mechanism of many chemical reactions and biochemical transformations, including those involving specific interactions with proteins, enzymes, and receptors.

**Fig. 1:**
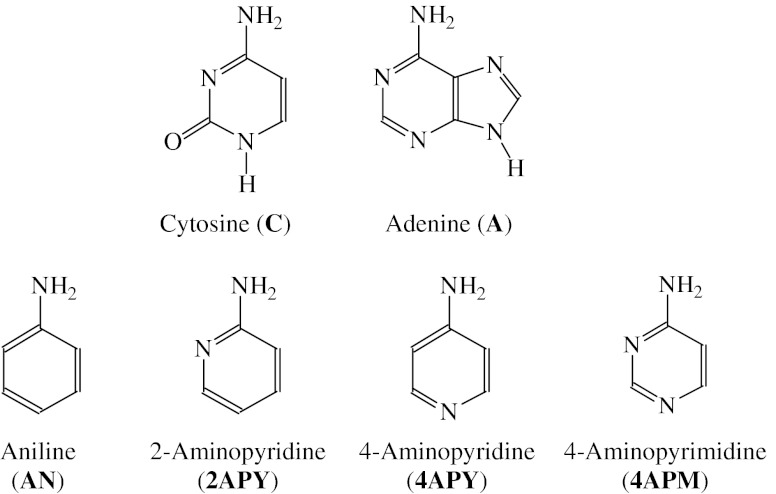
Nucleobases with the NH_2_ group and their convenient models

To understand prototropic tautomerism for nucleobases possessing the exo NH_2_ group (cytosine and adenine given in Fig. 1), it is very important to understand first the tautomeric conversions for their model compounds. For our investigations, we chose the neutral and unpaired ionic forms of convenient models – six membered rings with the exo NH_2_ group and the endo N-aza atom(s). In our previous papers, we studied the enamine-imine conversions for the neutral and redox forms of aniline (**AN**) [6] and the amine-imine and enamine-imine conversions for the neutral and redox forms of 4-aminopyrimidine (**4APM**) [7]. We found an interesting change of the tautomeric preference for the unpaired anions. To complete these studies for model aminoazines and to analyze effects of the N-aza group(s), we chose 2- (**2APY**) and 4-aminopyridines (**4APY**). We applied the same methods as previously described for **AN** and **4APM** [6, 7].

In the literature, one can find numerous papers for neutral aminopyridines. For example, tautomeric equilibria for their methyl derivatives have been theoretically studied using the DFT, PCM and SCI-PCM methods [8]. The amine form strongly dominates in the gas phase as well as in solution. The fixed imine form {2(1H)-pyridinimine} has been investigated for its 1-methyl derivative [9–13]. Alkorta and Elguero analyzed the dimerization of **2APY** by the hydrogen bond formation and studied the dimerization effect on tautomeric equilibria using *ab initio* methods [14]. They also discussed the substituent effects on the tautomeric preferences. Interesting studies have been carried out by chemists from Japan and Taiwan. They studied the hydrogen bonding and the imine form formation for the **2APY**/acetic acid system in the ground and excited state using various spectroscopic and quantum-chemical methods [15–20]. Photoinduced reversible amine-imine tautomerism has also been detected for **2APY** and for its derivatives by Ar-matrix-isolation infrared spectroscopy and DFT calculations [21–23]. To our knowledge, there is no report on prototropy for redox forms of **2APY** and **4APY**, which may be formed in the presence of various oxidizing and reducing agents.

In this paper, we studied the consequences of one-electron oxidation and one-electron reduction on tautomeric equilibria and on composition of the tautomeric mixture for 2- (**2APY**) and 4-aminopyridine (**4APY**). We considered various oxidation states of aminopyridines, neutral (**2APY** and **4APY**), oxidized (**2APY** ^**+•**^ and **4APY** ^**+•**^), and reduced state (**2APY** ^**-•**^ and **4APY** ^**-•**^). To investigate variations of π-electron delocalization for the ring and for the whole tautomeric system, we analyzed the geometrical parameters of all possible tautomers for the neutral and ionic forms of aminopyridines. We discussed also the spin populations for heavy atoms in the ionic forms. We estimated the tautomeric equilibrium constants and the oxidation and reduction energies. Finally, we compared our results with those reported previously for aniline [6] and 4-aminopyrimidine [7], and we discussed effects of the N-aza group(s).

## Computational details

Geometries of all neutral and ionic tautomers of 2- and 4-aminopyridines were fully optimized without symmetry constraints employing the DFT(B3LYP) method [24–26] and the 6-311+G** basis set [27]. For all neutral, oxidized, and reduced isomers, the DFT minima were found with all real frequencies and thermodynamic parameters such as the energy (*E*), enthalpy (*H*), entropy (*S*), and Gibbs energy (*G* for *T* = 298.15 K) were calculated at the same level of theory. All calculations were performed using the Gaussian 03 program [28].

## Results and discussion

### Choice of methods

For our investigations on prototropy for the neutral and redox forms of aminopyridines, we chose quantum-chemical methods because experimental techniques are incapable of detecting less than 0.1 % of minor tautomer(s) [15–23]. Tautomeric conversions are very fast and reversible processes, and thus it is difficult to separate and to study individual tautomers. Applying spectroscopic techniques such as ultraviolet (UV), infrared (IR), Raman, nuclear magnetic resonance (NMR), microwave (MW), mass spectrometry (MS), etc. to tautomeric mixtures one may identify signals of significant intensities solely for major tautomers (NH forms). Minor tautomers (CH forms) cannot be detected, because their amounts are too small (< 0.1 %) and their signals are in the background.

We applied the DFT method [24] with the B3LYP functional [25, 26] and the 6-311+G(d,p) basis set with the diffuse and polarization functions [27]. The DFT method has been applied for proton transfer reactions, including the tautomeric conversions in the gas phase that models a polar environment [29–37]. However, it should be mentioned here that the B3LYP method should be very carefully used for tautomeric systems. In some cases, e.g., for amide-iminol equilibrium in 2-hydroxypyridine/2-pyridone, where the relative energies for the major tautomers are close to zero, the B3LYP predictions overestimate the experimental data [38]. Errors are slightly larger than 1 kcal mol^-1^. For this reason, we tested various levels of theory for aniline [6] – the parent system of azaanilines: B3LYP/6-311+G(d,p), B3LYP/6-311++G(3df,3pd), B3LYP/aug-cc-pVDZ, and G2, and we found that (i) the B3LYP/6-311+G(d,p) level is sufficient for the tautomeric systems of amino aromatics, (ii) the use of different basis set for DFT calculations has no important effect on the values of geometric and energetic parameters, and (iii) the DFT relative Gibbs energies are close to those at the G2 level, recommended for the proton-transfer reactions in the gas phase [39, 40].

Using the quantum-chemical methods, we can study all possible prototropic tautomers and all possible tautomeric conversions for various oxidation states of 2- and 4-aminopyridines: the neutral state (**APY**), the unpaired cation (**APY** − e → **APY** ^**+•**^), and the unpaired anion (**APY** + e → **APY** ^**-•**^). Transferring an electron from or to the tautomeric molecule may change the stabilities of individual tautomers, and consequently, the composition of the tautomeric mixture.

### Possible tautomeric equilibria

**Scheme 1:**
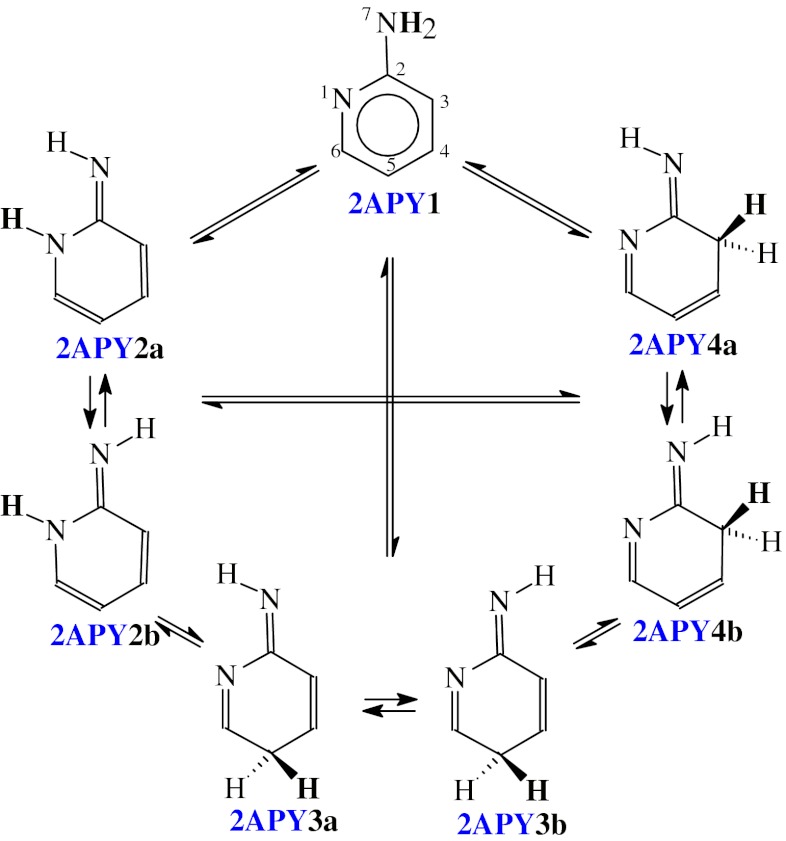
Tautomeric equilibria for **2APY** (labile proton marked in bold)

**Scheme 2:**
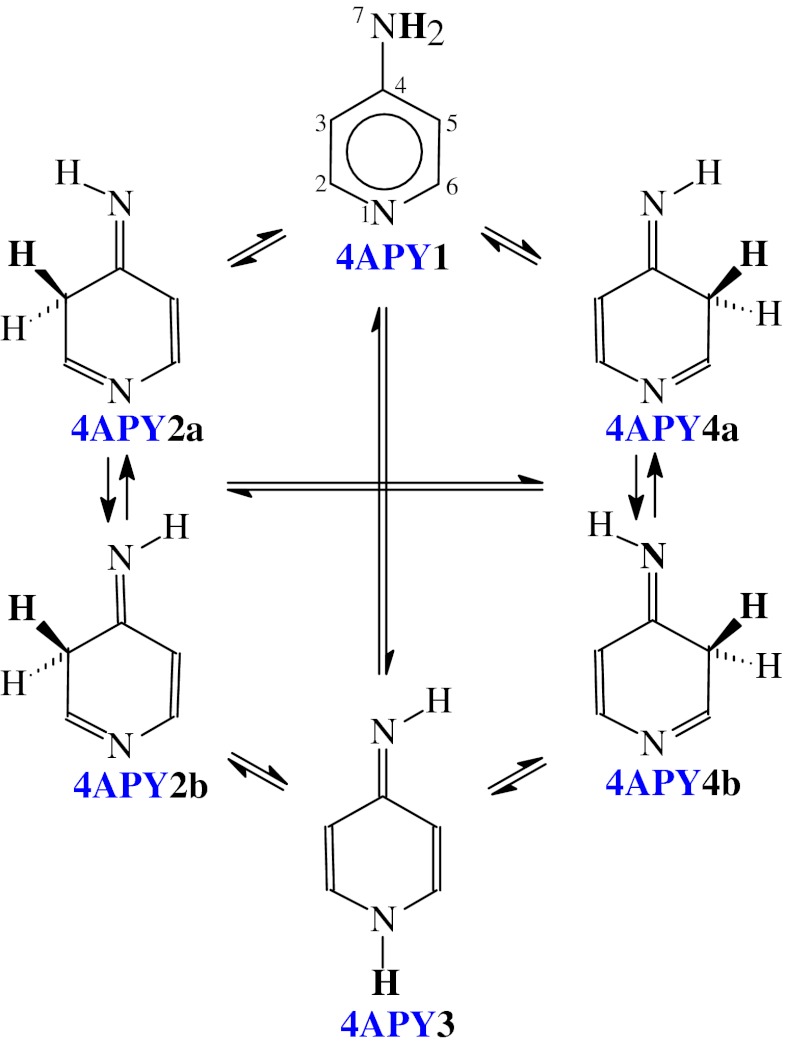
Tautomeric equilibria for **4APY** (labile proton marked in bold)

Aminopyridines **2APY** and **4APY** contain two functional groups, the exo NH_2_ group and the endo N-aza group which are n-π and π-π conjugated with the endo >C=C< groups. They exhibit prototropic tautomerism. One labile proton can move from the exo NH_2_ group to the endo N or C atom. Combination of two types of conversions: amine-imine {−NH−C(R)=N− → −N=C(R)−NH−} and enamine-imine {>C=C(R)−NH− → >CH−C(R)=N−} tautomerism leads to six tautomeric equilibria for four tautomers (Scheme 1 and 2), one amine form with the labile proton at the exo N atom, and three imine form with the labile proton at the endo N and C atoms for the NH and CH tautomers, respectively. For all tautomers, the intramolecular proton-transfer is accompanied by migration of π-electrons.

Due to geometric isomerism of the exo =NH group in 2-aminopyridine (Scheme 1), two isomers are possible for **2APY2-2APY4**, one (**a**) with the imine H atom synperiplanar to the ring N1 atom and the other one (**b**) with the imine H atom antiperiplanar to the ring N1 atom. Similarly, two isomers are possible for the imine forms **4APY2** and **4APY4** of 4-aminopyridine (Scheme 2), one (**a**) with the imine H atom synperiplanar to the ring C3 (for **4APY2**) and C5 (for **4APY4**) atom, and the other one (**b**) with the imine H atom antiperiplanar. The structures **4APY2a** and **4APY4a**, and also **4APY2b** and **4APY4b** are identical for 4-aminopyridine, and thus the tautomeric equilibrium constants *K*_T_ for the conversions **4APY2a** → **4APY4a** and **4APY2b** → **4APY4b** are equal to unity. However, to estimate well the composition of the tautomeric mixture, all isomers (**4APY1**-**4APY4**) should be considered for 4-aminopyridine. In the literature, four tautomers have been solely considered for the neutral forms of methyl derivatives of aminopyridines [8].

### DFT structures

For all isomers of neutral and ionic **2APY** and **4APY** (Scheme 1 and 2), the minima with all real frequencies were found at the B3LYP/6-311+G** level. As expected [6, 7], the exo NH_2_ group is planar solely for the unpaired cations **2APY1** ^**+•**^ and **4APY1** ^**+•**^. For neutral **2APY1** and **4APY1**, and for unpaired anions **2APY1** ^**-•**^ and **4APY1** ^**-•**^, this group takes the pyramidal conformation similar to that for the neutral and anionic forms of aniline and 4-aminopyrimidine [6, 7, 41, 42]. Our results confirm the nonplanarity of the exo NH_2_ group for neutral aminopyridines discussed earlier in the literature [43, 44]. Transfer of the proton to the endo N atom does not destroy the planarity of the ring for the neutral NH tautomers (**2APY2a**, **2APY2b**, and **4APY3**) and for their unpaired cations (**2APY2a** ^**+•**^, **2APY2b** ^**+•**^, and **4APY3** ^**+•**^). Some exceptions are the unpaired anions (**2APY2a** ^**-•**^, **2APY2b** ^**-•**^, and **4APY3** ^**-•**^) for which the endo N atom takes the pyramidal conformation. Due to presence of the C-sp^3^ atom (C3 and C5), all neutral CH isomers (**2APY3a**, **2APY3b**, **2APY4a**, **2APY4b**, **4APY2a/4a**, and **4APY2b/4b**), their unpaired cationic (**2APY3a** ^**+•**^, **2APY3b** ^**+•**^, **2APY4a** ^**+•**^, **2APY4b** ^**+•**^, **4APY2a/4a** ^**+•**^, and **4APY2b/4b** ^**+•**^) and anionic structures (**2APY3a** ^**-•**^, **2APY3b** ^**-•**^, **2APY4a** ^**-•**^, **2APY4b** ^**-•**^, **4APY2a/4a** ^**-•**^, and **4APY2b/4b** ^**-•**^) lose their planarity.

**Fig. 2:**
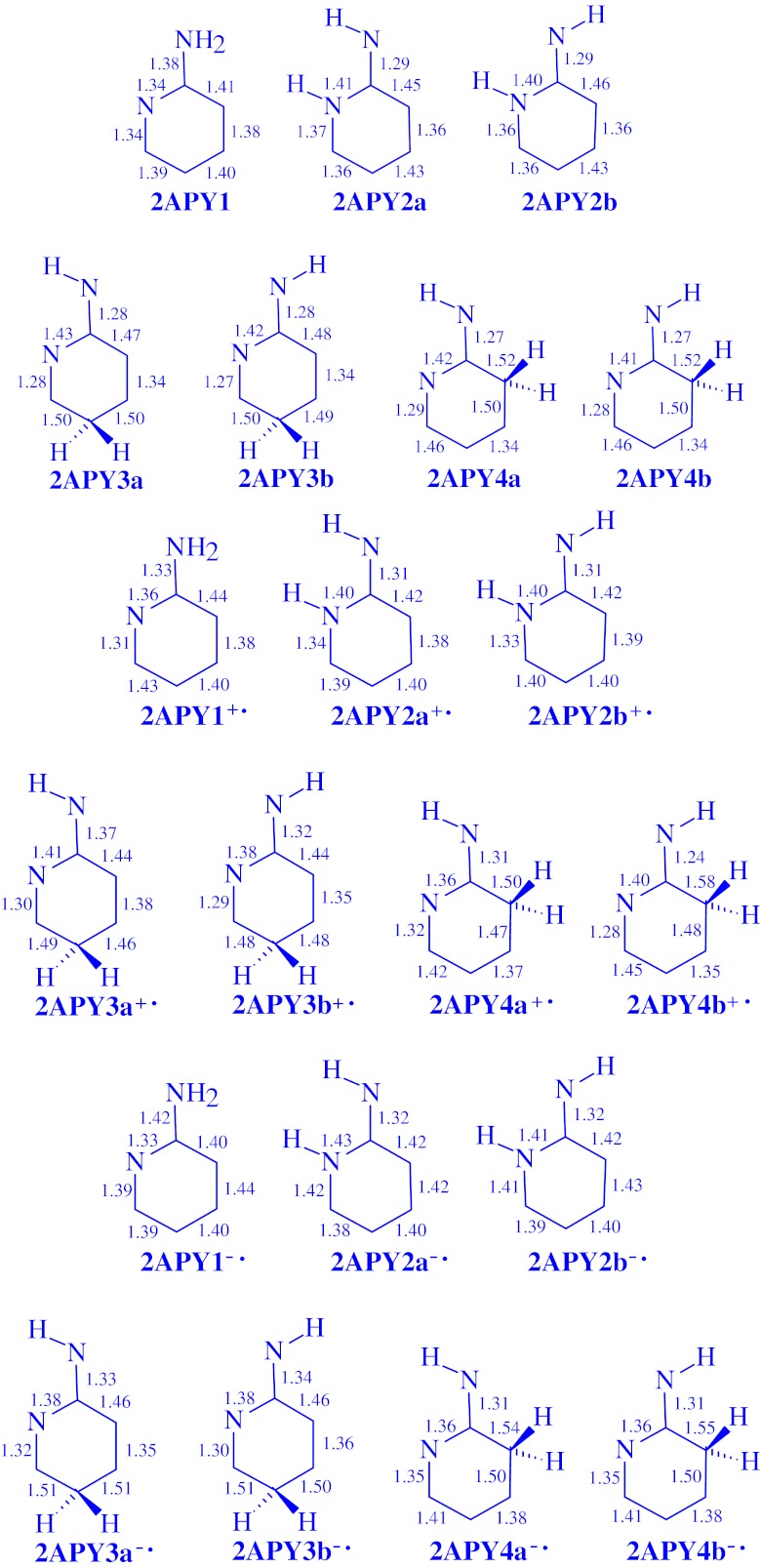
The DFT-calculated CC and CN bond lengths (in Å) for isomers of neutral **2APY** and its unpaired ions **2APY** ^**+•**^ and **2APY** ^**-•**^

**Fig. 3:**
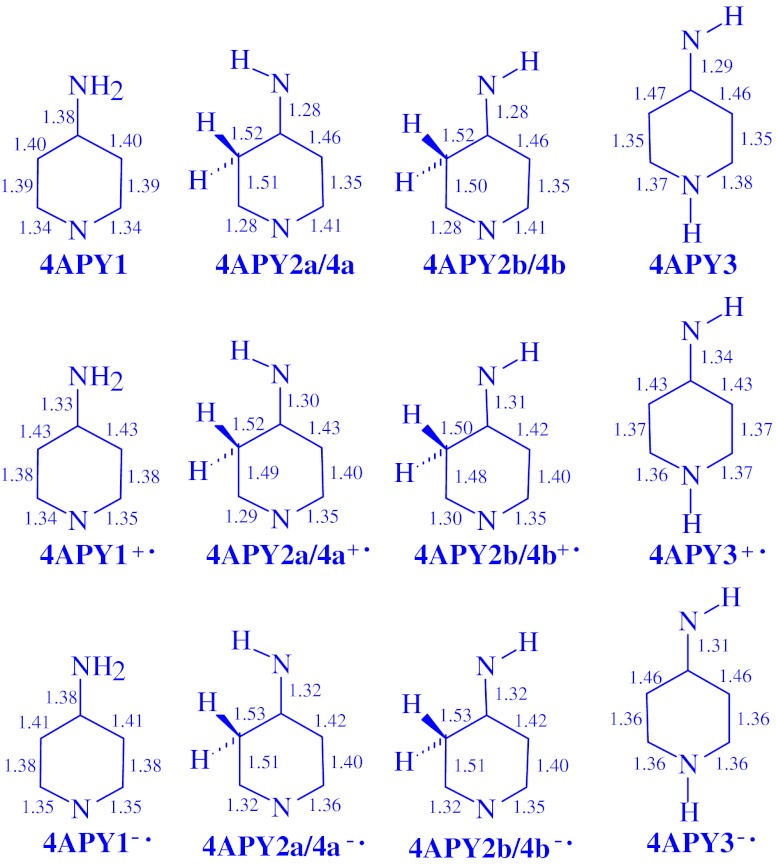
The DFT-calculated CC and CN bond lengths (in Å) for isomers of neutral **4APY** and its unpaired ions **4APY** ^**+•**^ and **4APY** ^**-•**^

The DFT-calculated CC and CN bond lengths for the neutral and redox isomers of 2- and 4-aminopyridines are given in Figs. 2 and 3, respectively. For neutral **2APY1, 2APY2a**, and **2APY2b**, they are close to those calculated by Akai et al. [21] at the B3LYP/6-31++G(d,p) level. Differences in bond lengths are not larger than 0.01 Å. Variations of the CC and CN bond lengths for the neutral and charged isomers of aminopyridines are also similar to those previously observed for aniline [6] and 4-aminopyrimidine [7]. Generally, the CC bond lengths for **2APY** and **4APY** vary from 1.34 to 1.52 Å for the neutral isomers, from 1.35 to 1.58 Å for the unpaired cations, and from 1.35 to 1.55 Å for the unpaired anions. Variations of the CN bond lengths are as follows: 1.27-1.43, 1.24-1.41, 1.30-1.43 Å, respectively.

When proceeding from the neutral isomers to their charged ions, the CC and CN bond lengths vary by 0.0-0.9 Å for the radical cations and by 0.0-0.7 Å for the radical anions. These variations influence π-electron delocalization in the ring (six atoms) and also in the whole tautomeric system (seven atoms including the exo N atom). In this paper, we quantitatively measured the changes of π-electron delocalization by means of the geometry based harmonic oscillator model of electron delocalization (HOMED) index [6, 7, 35, 45–48] which describes well any type of conjugation (π-π, n-π, and σ-π) possible in heteroatomic systems. This index was applied here to the DFT-calculated bond lengths.

## HOMED indices

The HOMED procedure has been described in detail in ref [46] and applied to various cyclic and acyclic π-electron systems containing heteroatoms [6, 7, 35, 45–48]. The HOMED index is based on the original harmonic oscillator model of aromaticity (HOMA) index [49, 50]. The HOMA index reformulated in 1993 [51] seems to be inappropriate for π-electron delocalized systems containing heteroatoms. The main reason is a use of different measures of π-electron delocalization for the reference CC and CX bonds [6, 7, 35, 45–48]. The reformulated HOMA index can be solely applied for homoaromatics. Its application to heteroaromatics leads to artificial positive or negative values for well delocalized π-electron systems [46].

The HOMED index can be estimated on the basis of the theoretically derived bond lengths using the following equation:${\text{HOMED}} = {1} - \left[ {\alpha \cdot \Sigma {{\left( {{R_{\text{o}}} - {R_{\text{i}}}} \right)}^2}} \right]:n$ [45, 46]. This equation is similar to that for the reformulated HOMA index [51], but the values of its parameters (α and *R*_o_) are different. In this equation, α is a normalization constant, *R*_o_ is the optimum bond length (assumed to be realized for fully delocalized system), *R*_i_ are the running bond lengths in the system, and *n* is the number of bonds taken into account. The following *R*_o_ values (in Å), calculated at the B3LYP/6-311+G(d,p) level [46], were taken here: 1.394 (benzene) and 1.334 (1,3,5-triazine) for the CC and CN bonds, respectively. Similarly as in the case of aniline [6], 4-aminopyrimidine [7], and other heteroaromatic molecules [46], the normalization α constants equal to 88.09 (CC) and 91.60 (CN) were used for the ring (six bonds), and 80.90 (CC) and 84.52 (CN) for the whole tautomeric system (seven bonds including the exo N atom). The α constants differ from those employed for the reformulated HOMA index, because of use of simple only slightly delocalized reference CC (ethane and ethene [46] instead of 1,3-butadiene [51]) and CN bonds (methylamine and methylimine [46, 51]), and different procedures for the even and odd number of bonds in the system [6, 7, 45, 46]. For estimation of the α constants, the reference CC and CN bond lengths were calculated at the B3LYP/6-311+G(d,p) level.

**Fig. 4:**
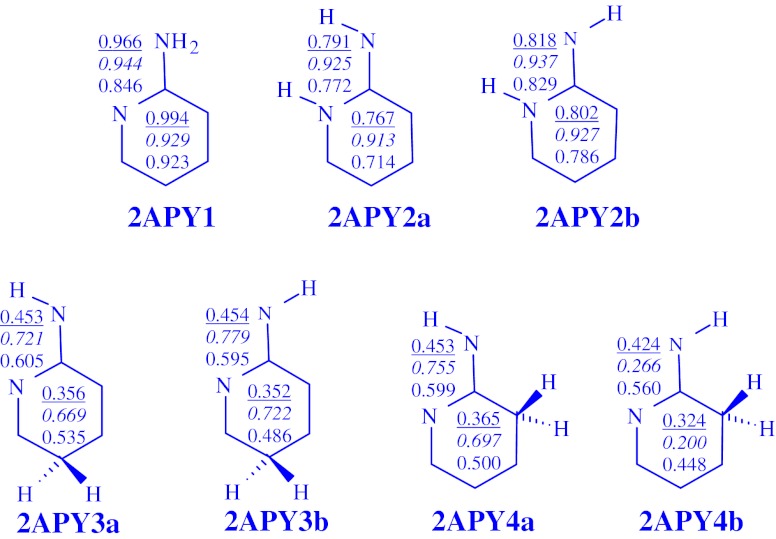
The HOMED indices estimated for the DFT-structures of neutral (underlined values), oxidized (values in italic style), and reduced forms (values in normal style) of **2APY** for the ring (six bonds, values included in the ring) and for the whole tautomeric system (seven bonds, values placed near the formula)

**Fig. 5:**
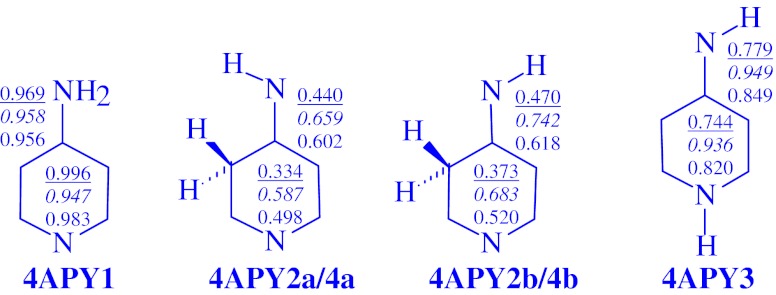
The HOMED indices estimated for the DFT-structures of neutral (underlined values), oxidized (values in italic style), and reduced forms (values in normal style) of **4APY** for the ring (six bonds, values included in the ring) and for the whole tautomeric system (seven bonds, values placed near the formula)

Similarly as for aniline [6] and 4-aminopyrimidine [7], the HOMED indices, estimated for the neutral isomers, are close to unity for the amine tautomers **2APY1** (Fig. 4) and **4APY1** (Fig. 5). Due to cross π-π and n-π conjugations possible in the whole tautomeric system, i.e., conjugation of n-electrons of the exo NH_2_ group with π-electrons of the ring, the HOMED indices decrease when going from the ring (six bonds) to the whole tautomeric system (seven bonds). Transfer of the proton to the endo N and C atom decreases the HOMED indices by 0.2-0.3 and 0.6-0.7 units, respectively. The imine NH isomers (**2APY2a**, **2APY2b**, and **4APY3**) are less delocalized than the amine tautomers (**2APY1** and **4APY1**), but more delocalized than the imine CH isomers (**2APY3a**, **2APY3b**, **2APY4a**, **2APY4b, 4APY2a/4a**, and **4APY2b/4b**). The N1 atom taking the labile proton retains its planarity in the neutral form due to n-π conjugation similar to that for the N1 atom in the five membered ring of pyrrole, pyrazole, imidazole, etc., whereas the C3 and C5 atoms (C-sp^3^), taking the labile proton, lose their planarity. For the imine CH isomers, π-electrons of the π-π conjugated −C=N−C=N−C=NH fragment are cross hyperconjugated with σ-electrons of the CH_2_ group. Usually, σ-π hyperconjugation leads to smaller electron delocalization than π-π and n-π conjugations [46]. Thus, the imine NH tautomers are still aromatic but the imine CH tautomers already have nonaromatic character.

One-electron oxidation and also one-electron reduction decrease aromaticity of the amine tautomers (**2APY1** and **4APY1**). The HOMED indices are reduced by less than 0.1 units. On the other hand, one-electron oxidation increases the HOMED indices for the imine NH tautomers (**2APY2a**, **2APY2b**, and **4APY3**) by 0.1-0.2 units increasing also their aromatic character. One-electron reduction has a slight effect. It changes the HOMED indices for the NH tautomers by less than ±0.1 units. Interestingly, for the imine CH tautomers (**2APY3a**, **2APY3b**, **2APY4a**, **2APY4b, 4APY2a/4a**, and **4APY2b/4b**), both one-electron oxidation and one-electron reduction increase the HOMED indices, but by different degree (by 0.2-0.4 and 0.1-0.2 units, respectively). For some cationic CH isomers (e.g., **2APY3a** ^**+•**^, **2APY3b** ^**+•**^, **2APY4a** ^**+•**^, and **4APY2b/4b** ^**+•**^), the HOMED indices for the whole tautomeric system (seven bonds) are even close to 0.8, indicating exceptional π-electron delocalization.

### Spin densities

**Fig. 6:**
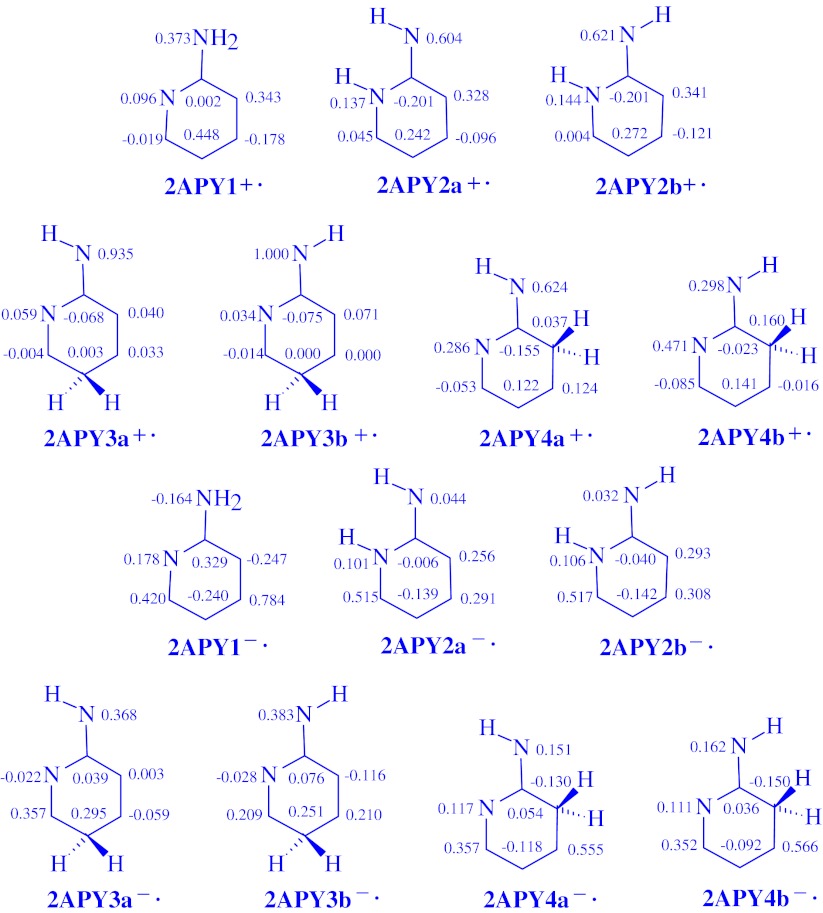
The DFT atomic spin populations for seven heavy atoms present in the ionic forms of **2APY**, the radical cations (**2APY** ^**+•**^) and the radical anions (**2APY** ^**-•**^)

**Fig. 7:**
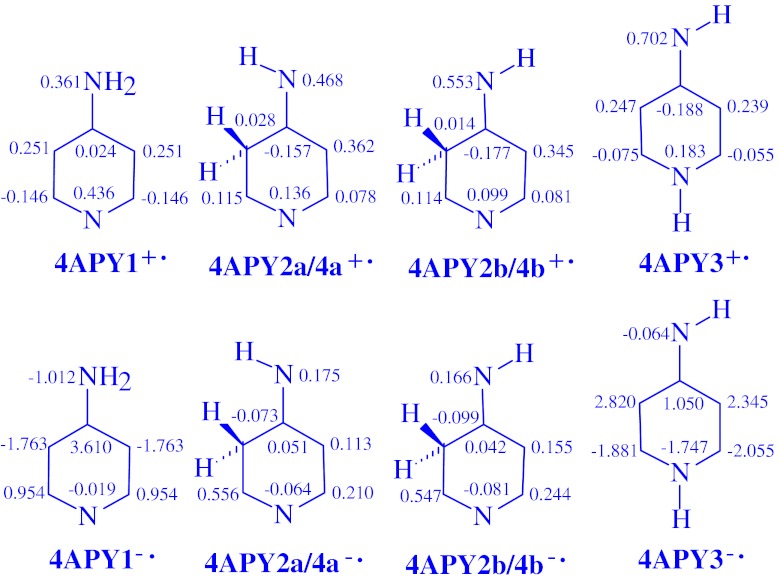
The DFT atomic spin populations for seven heavy atoms present in the ionic forms of **4APY,** the radical cations (**4APY** ^**+•**^) and the radical anions (**4APY** ^**-•**^)

Some additional information on delocalization of one unpaired electron may be derived on the basis of the distribution of the unpaired spin density calculated for the charged radicals of aminopyridines. The total atomic spin densities calculated at the DFT/B3LYP/6-311+G(d,p) level for seven heavy atoms in **2APY** ^**+•**^ and **2APY** ^**-•**^, also in **4APY** ^**+•**^ and **4APY** ^**-•**^ are given in Figs. 6 and 7, respectively. Unfortunately, there is no experimental data for the aminopyridine charged radicals and no comparison can be made. Our DFT calculations indicate that the spin density exists on all atoms of the charged radicals. In the case of the amine radical cations **2APY1** ^**+•**^ and **4APY1** ^**+•**^, most of the density is carried by the N1 and N7 atoms, and also by the C3 and C5 atoms. On the C2, C4, and C6 atoms, a spin population is also present, but it is negative or close to zero. For the amine radical anions **2APY1** ^**-•**^ and **4APY1** ^**-•**^, most of the density is localized on the C2, C4, and C6 atoms. On the other atoms, a spin population also exists, but it is considerably lower or negative.

For the imine tautomers, the distribution of the spin density depends on the position of the labile proton and charge. In the case of the imine NH radical cations **2APY2** ^**+•**^ and **4APY3** ^**+•**^, most of the spin density is carried by the N7, N1, C3, and C5 atoms. For the other C atoms the spin density values are negative or close to zero. For the imine NH radical anions, the C6, C4, and C3 atoms take most of the spin density in **2APY2** ^**-•**^, and the C3, C5, and C4 atoms in **4APY3** ^**-•**^. A spin population also exists on the N1 and N7 atoms in **2APY2** ^**-•**^, but it is considerably lower. On the other atoms, the spin density values are negative. For the imine CH radical cations, most of the spin density is located on the N7 atom in **2APY3** ^**+•**^, on the N7, N1, C3, or C5 atom in **2APY4** ^**+•**^, and on the N7, N1, C5, and C2 atom in **4APY2** ^**+•**^. For the imine CH radical anions, most of the spin density is taken by the N7, C5, and C6 atoms in **2APY3** ^**-•**^, by the C4, C6, N7, and N1 atoms in **2APY4** ^**-•**^, and by the C2, C5, C6, and N7 atoms in **4APY2** ^**-•**^. For the other atoms, the spin density values are negative or close to zero.

### Tautomeric preferences

As expected [6–8, 14–23], the amine tautomers **2APY1** and **4APY1** with the labile proton at the exo N atom has the lowest energy for neutral aminopyridines. Similar to aniline [6] and 4-aminopyrimidine [7], aromaticity of the six membered ring seems to be one of the most important factors that dictates the high stability of the neutral amine tautomers and their tautomeric preference (100 %) in the tautomeric mixture. Tables 1 and 2 summarize the relative thermodynamic parameters {estimated at the B3LYP/6-311+G(d,p) level} such as the relative energies (Δ*E*_T_), the relative enthalpies (Δ*H*_T_), the relative entropy terms (*T*Δ*S*_T_), the relative Gibbs energies (Δ*G*_T_), the tautomeric equilibrium constants (as p*K*_T_ = Δ*G*_T_/2.303R*T*), and the percentage contents (*x* = *K*_T_/(1 + *K*_T_)} of all individual neutral and ionic tautomers of 2- and 4-aminopyridines, respectively. The Δ*G* values include changes in the electronic energy, in the zero-point energy (ZPE), and in the thermal corrections to the energy and entropy (vibrational, rotational, and translational).

**Table 1:** The DFT-calculated relative thermodynamic parameters (Δ*E*_T_, Δ*H*_T_, *T*Δ*S*_T_, Δ*G*_T_, and p*K*_T_) and the percentage contents (*x* in %) for the neutral isomers of **2APY** and for its unpaired ions **2APY** ^**+•**^ and **2APY** ^**-•**^

Isomer	Charge	Δ*E*_T_^a,b^	Δ*H*_T_^b,c^	*T*Δ*S*_T_^b,c^	Δ*G*_T_^b,c^	p*K*_T_^c^	*x*
a) neutral^d^							
**2APY1**	0	0.0	0.0	0.0	0.0	0.0	100
**2APY2a**	0	16.7	16.7	0.0	16.7	12.2	6·10^-11^
**2APY2b**	0	14.0	13.9	0.0	13.9	10.2	6·10^-9^
**2APY3a**	0	29.2	29.2	0.4	28.8	21.1	8·10^-20^
**2APY3b**	0	33.4	33.4	0.6	32.8	24.0	9·10^-23^
**2APY4a**	0	30.7	30.7	0.4	30.4	22.2	6·10^-21^
**2APY4b**	0	35.4	35.4	0.4	35.0	25.7	2·10^-24^
b) oxidized^e^							
**2APY1** ^**+•**^	1	0.0	0.0	0.0	0.0	0.0	99.9
**2APY2a** ^**+•**^	1	7.4	7.4	−0.2	7.5	5.5	3·10^-4^
**2APY2b** ^**+•**^	1	3.8	3.6	−0.2	3.9	2.8	0.1
**2APY3a** ^**+•**^	1	50.6	50.9	0.8	50.2	36.8	2·10^-35^
**2APY3b** ^**+•**^	1	52.0	52.5	1.2	51.2	37.6	3·10^-36^
**2APY4a** ^**+•**^	1	50.2	49.9	−0.5	50.4	36.9	8·10^-30^
**2APY4b** ^**+•**^	1	53.4	53.8	0.9	52.9	38.8	2·10^-37^
c) reduced^f^							
**2APY1** ^**-•**^	−1	3.9	4.1	−0.1	4.2	3.1	8·10^-2^
**2APY2a** ^**-•**^	−1	9.4	9.7	0.2	9.5	7.0	1·10^-5^
**2APY2b** ^**-•**^	−1	6.1	6.4	0.2	6.2	4.5	3·10^-3^
**2APY3a** ^**-•**^	−1	11.9	11.8	−0.3	12.1	8.9	1·10^-9^
**2APY3b** ^**-•**^	−1	15.5	15.4	−0.3	15.8	11.5	3·10^-10^
**2APY4a** ^**-•**^	−1	0.0	0.0	0.0	0.0	0.0	99.9
**2APY4b** ^**-•**^	−1	4.8	4.8	0.0	4.8	3.5	3·10^-2^

^a^ ΔZPE included ^b^ In kcal mol^-1 c^ At 298.15 K ^d^ Thermodynamic parameters relative to those for **2APY1** ^e^ Thermodynamic parameters relative to those for **2APY1** ^**+•** f^ Thermodynamic parameters relative to those for **2APY4a** ^**-•**^

**Table 2:** The DFT-calculated relative thermodynamic parameters (∆*E*_T_, ∆*H*_T_, *T*∆*S*_T_, ∆*G*_T_, and p*K*_T_) and the percentage contents (x in %) for the neutral isomers of **4APY** and for its unpaired ions **4APY** ^**+•**^ and **4APY** ^**-•**^

Isomer	Charge	Δ*E*_T_^a,b^	Δ*H*_T_^b,c^	*T*Δ*S*_T_^b,c^	Δ*G*_T_^b,c^	p*K*_T_^c^	*x*
a) neutral^d^							
**4APY1**	0	0.0	0.0	0.0	0.0	0.0	100
**4APY2a/4a**	0	30.9	31.0	0.6	30.4	22.3	5·10^-21^
**4APY2b/4b**	0	30.3	30.4	0.6	29.8	21.8	1·10^-20^
**4APY3**	0	16.5	16.5	0.1	16.4	12.0	9·10^-11^
b) oxidized^e^							
**4APY1** ^**+•**^	1	1.8	1.8	−0.1	1.9	1.4	4.3
**4APY2a/4a** ^**+•**^	1	42.3	42.7	1.0	41.7	30.5	3·10^-29^
**4APY2b/4b** ^**+•**^	1	42.4	42.9	1.2	41.7	30.6	3·10^-29^
**4APY3** ^**+•**^	1	0.0	0.0	0.0	0.0	0.0	95.7
c) reduced^f^							
**4APY1** ^**-•**^	−1	4.1	4.0	−0.5	4.6	3.3	4·10^-2^
**4APY2a/4a** ^**-•**^	−1	1.1	1.1	−0.1	1.2	0.9	13.2
**4APY2b/4b** ^**-•**^	−1	0.0	0.0	0.0	0.0	0.0	86.8
**4APY3** ^**-•**^	−1	12.2	11.8	−1.0	12.9	9.4	4·10^-8^

^a^ ΔZPE included ^b^ In kcal mol^-1 c^ At 298.15 K ^d^ Thermodynamic parameters relative to those for **4APY1** ^e^ Thermodynamic parameters relative to those for **4APY3** ^**+•**^

^f^ Thermodynamic parameters relative to those for **4APY2b/4b** ^**-•**^

The neutral imine NH tautomers **2APY2** and **4APY3** possessing the labile proton at the endo N atom have larger energies than **2APY1** and **4APY1** by 14–17 kcal mol^-1^ at the B3LYP/6-311+G(d,p) level. Energetically favorable and energetically unfavorable interactions possible for the structures **a** and **b** of the **2APY2** tautomer differentiate their energies by only 3 kcal mol^-1^. Slightly larger energy differences (4–5 kcal mol^-1^) occur for the structures **a** and **b** of the imine CH tautomers **2APY3** and **2APY4**. In the case of the imine CH tautomers **4APY2/4**, this energy difference is not larger than 1 kcal mol^-1^. However, transfer of the labile proton to the endo C atom exceptionally decreases the stability of both isomers (**a** and **b**) of **2APY3**, **2APY4**, and **4APY2/4**. Their energies are larger than that of **2APY1** and **4APY1** by 29–35 kcal mol^-1^. The contributions of all imine NH and CH isomers in the tautomeric mixture of **2APY** and **4APY** are very low (< 1⋅10^-8^ %).

According to the DFT results, one-electron oxidation has no important effect on the tautomeric preference in the gas phase for 2-aminopyridine (Table 1). The amine tautomer **2APY1** ^**+•**^ has the lowest energy. However, oxidation changes the relative energies for the imine tautomers, and consequently, it changes also the composition of the tautomeric mixture. For the imine NH tautomer, the relative Gibbs energy decreases to 4 kcal mol^-1^ for **2APY2b** ^**+•**^ with energetically favorable configurations and to 8 kcal mol^-1^ for **2APY2a** ^**+•**^ with energetically unfavorable configurations. The reduction of the relative Gibbs energies for the NH isomers augments their contributions in the tautomeric mixture of **2APY** ^**+•**^. The percentage contents of **2APY2b** ^**+•**^ and **2APY2a** ^**+•**^ are considerably larger (0.1 and 3⋅10^-4^ %, respectively) than those in neutral **2APY** (< 1⋅10^-8^ %). They cannot be neglected in the tautomeric mixture of **2APY** ^**+•**^. Quite a different situation takes place for the imine CH tautomers. The relative Gibbs energies increase to more than 50 kcal mol^-1^ for both isomers (**a** and **b**) of **2APY3** ^**+•**^ and **2APY4** ^**+•**^. Change of the configuration for the exo =NH group has a small effect on the relative Gibbs energies (< 3 kcal mol^-1^). The percentage contents of the imine CH isomers are very low (< 1·10^-29^ %). As very rare isomers, they may be neglected in the tautomeric mixture of **2APY** ^**+•**^.

In the case of 4-aminopyridine, one-electron oxidation also changes the DFT-stabilities of the amine **4APY1** ^**+•**^ and imine **4APY3** ^**+•**^ tautomers (Table 2) but in higher degree than for 2-aminopyridine (Table 1). Consequently, **4APY3** ^**+•**^, instead of **4APY1** ^**+•**^, seems to be the favored tautomer for the radical cation. Since the relative Gibbs energy between the two tautomers is not very large (ca. 2 kcal mol^-1^), they may dictate the composition of the tautomeric mixture (95.7 and 4.3 %, respectively). The relative Gibbs energies of the imine CH isomers **4APY2a/4a** ^**+•**^ and **4APY2b/4b** ^**+•**^ are larger than 40 kcal mol^-1^ and they seem to not depend on the configuration of the exo =NH group. The two CH isomers may be neglected in the tautomeric mixture of **4APY** ^**+•**^. Their percentage contents are exceptionally low (< 1·10^-28^ %).

One-electron reduction changes the tautomeric preference for both 2- and 4-aminopyridines. For reduced aminopyridines, the imine CH isomers **2APY4a** ^**-•**^ and **4APY2b/4b** ^**-•**^ with the labile proton at the endo C3/C5 atom and with the imine H atom antiperiplanar to the C3/C5 atom have the lowest energies at the B3LYP/6-311+G(d,p) level (Tables 1 and 2). The **2APY4b** ^**-•**^ and **4APY2a/4a** ^**-•**^ isomers with the imine H atom synperiplanar to the C3/C5 atom possess larger Gibbs energies than **2APY4a** ^**-•**^ and **4APY2b/4b** ^**-•**^ by ca. 5 and 1 kcal mol^-1^, respectively. The other reduced tautomers have larger Gibbs energies than those of **2APY4a** ^**-•**^ and **4APY2b/4b** ^**-•**^ by 4–16 kcal mol^-1^. The tautomeric mixture of **2APY** ^**-•**^ seems to consist mainly of **2APY4a** ^**-•**^ (99.9 %) and that of **4APY** ^**-•**^ contains **4APY2b/4b** ^**-•**^ and **4APY2a/4a** ^**-•**^ (86.8 and 13.2 %, respectively). The percentage contents of the other isomers are lower than 0.1 %.

**Scheme 3:**
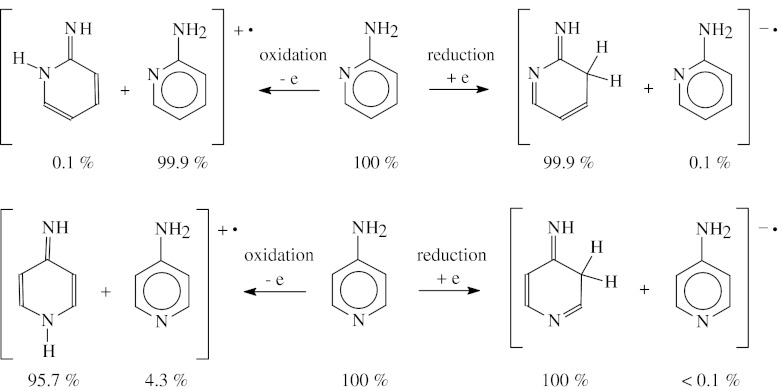
The tautomeric preferences for the redox forms of aminopyridines estimated at the DFT(B3LYP)/6-311+G(d,p) level

If we consider solely the DFT-favored neutral and redox forms of 2- and 4-aminopyridines, the following scheme of the redox reactions can be drawn (Scheme 3). The percentage contents of the major amine and imine tautomers are also given in this scheme. Generally, the tautomeric preferences for aminopyridines are the same for the neutral and reduced forms. The neutral tautomeric mixtures of **2APY** and **4APY** contain mainly the amine tautomers (100 %), and those of **2APY** ^**-•**^ and **4APY** ^**-•**^ consists of the major imine CH tautomers (99.9-100 %) and of the minor amine tautomers (≤ 0.1 %). Solely, the tautomeric preferences for the oxidized forms seem to be dependent on the positions of the exo and endo N atoms. The amine tautomer (99.9 %) is favored for **2APY** ^**+•**^ whereas the imine NH tautomer (95.7 %) dominates for **4APY** ^**+•**^.

Since the relative energies between the major tautomers of the ionic forms of **4APY** are only slightly larger than the error of the B3LYP method estimated for 2-hydroxypyridine/2-hydroxypyridone (ca. 1 kcal mol^-1^) [38], the DFT results should be very carefully used for interpretation of oxidation and reduction reactions. A slight change of environment may change the relative energy values, and consequently, it may change the composition of the tautomeric mixture.

The relative entropy terms (*T*Δ*S*_T_) for the neutral and redox forms of 2- and 4-aminopyridines are not larger than ±1 kcal mol^-1^. This may suggest that all tautomeric conversions are isoentropic in the gas phase and do not depend on the oxidation or reduction state of the molecule. Generally, there are not large structural changes during tautomerization. Some exceptions are those resulting from loss of the molecule planarity. The relative thermal corrections (from zero to 298.15 K) are close to zero, and thus, Δ*E*_T_ ≈ Δ*H*_T_ ≈ Δ*G*_T_. All these observations suggest that the relative thermodynamic parameters do not depend very much on temperature (thermal corrections and entropy terms cancel out), and thus the percentage contents of the same order of magnitude may be expected for individual tautomers in a jet-cooled experiments. Very low thermal corrections and entropy terms have been also observed for aniline [6] and 4-aminopyrimidine [7].

### Oxidation and reduction energies

For each isomer of **2APY** and **4APY**, thermodynamic parameters of oxidation (Δ*E*_ox_, Δ*H*_ox_, *T*Δ*S*_ox_, and Δ*G*_ox_) and reduction (Δ*E*_red_, Δ*H*_red_, *T*Δ*S*_red_, and Δ*G*_red_) were estimated at the DFT(B3LYP)/6-311+G(d,p) level. They were calculated as differences between the corresponding parameters for the charged and neutral forms. First perusal of these parameters indicates that one-electron oxidation is a very endothermic process (Table 3). The lowest energies (173–174 kcal mol^-1^) are needed for the imine NH tautomers, and the largest energies (201–205 kcal mol^-1^) for the imine CH tautomers. For the amine tautomers, the oxidation Gibbs energies (Δ*G*_ox_ 183–192 kcal mol^-1^) are between those for the imine NH and CH tautomers. Change of the configuration of the imine H atom in structures **a** and **b** has small effect on the Δ*G*_ox_ values (< 3 kcal mol^-1^). Interestingly, the literature ionization energies for 2-aminopyridine (8.5 eV [52], 1 eV = 23.06037 kcal mol^-1^) and for 4-aminopyridine (8.8 eV [52]) are of the same order of magnitude as the DFT estimated energies of oxidation.

**Table 3:** The DFT-estimated energies of oxidation (Δ*E*_ox_, Δ*H*_ox_, and Δ*G*_ox_) and entropy terms (*T*Δ*S*_ox_) for individual isomers of **2APY** and **4APY**

Oxidation	Δ*E*_ox_^a,b^	Δ*H*_ox_^b,c^	*T*Δ*S*_ox_^b,c^	Δ*G*_ox_^b,c^
**2APY1** − e → **2APY1** ^**+•**^	183.7	183.7	0.4	183.3
**2APY2a** − e → **2APY2a** ^**+•**^	174.4	174.4	0.2	174.1
**2APY2b** − e → **2APY2b** ^**+•**^	173.5	173.4	0.2	173.2
**2APY3a** − e → **2APY3a** ^**+•**^	205.2	205.4	0.7	204.7
**2APY3b** − e → **2APY3b** ^**+•**^	202.3	202.8	1.0	201.8
**2APY4a** − e → **2APY4a** ^**+•**^	203.3	203.7	1.2	202.5
**2APY4b** − e → **2APY4b** ^**+•**^	201.8	202.1	0.9	201.2
**4APY1** − e → **4APY1** ^**+•**^	192.0	192.0	0.5	191.6
**4APY2a** − e → **4APY2a** ^**+•**^	201.2	201.4	0.6	200.8
**4APY2b** − e → **4APY2b** ^**+•**^	201.9	202.2	0.8	201.4
**4APY3** − e → **4APY3** ^**+•**^	173.4	173.2	0.2	173.1

^a^ ΔZPE included ^b^ In kcal mol^-1 c^ At 298.15 K

One-electron reduction is a more favorable process than one-electron oxidation. Comparison of the DFT calculated thermodynamic parameters for the reduced and neutral tautomers of **2APY** and **4APY** shows that one-electron reduction requires less than 20 kcal mol^-1^ (Table 4). For the imine CH isomers, the reduction Gibbs energies (Δ*G*_red_) are even negative, indicating that aminopyridines may take spontaneously one electron from a reducing agent. They may be more easily transformed to the reduced forms than the imine NH and amine tautomers for which the Δ*G*_red_ values are positive. This trend confirms preference of the imine CH tautomers in the tautomeric mixtures of **2APY** ^**-•**^ and **4APY** ^**-•**^. Change of the position of the imine H atom has no important effect on the Δ*G*_red_ values (< 1 kcal mol^-1^). There are no experimental data in the literature [53] for the electron affinity of **2APY** and **4APY** and no comparison can be made.

**Table 4:** The DFT-estimated energies of reduction (Δ*E*_red_, Δ*H*_red_, and Δ*G*_red_) and entropy terms (*T*Δ*S*_red_) for individual isomers of **2APY** and **4APY**

Reduction	Δ*E*_red_^a,b^	Δ*H*_red_^b,c^	*T*Δ*S*_red_^b,c^	Δ*G*_red_^b,c^
**2APY1** − e → **2APY1** ^**-•**^	17.6	16.1	1.0	15.1
**2APY2a** − e → **2APY2a** ^**-•**^	4.5	5.0	1.3	3.7
**2APY2b** − e → **2APY2b** ^**-•**^	3.9	4.5	1.3	3.2
**2APY3a** − e → **2APY3a** ^**-•**^	−5.5	−5.4	0.4	−5.8
**2APY3b** − e → **2APY3b** ^**-•**^	−6.1	−6.0	0.1	−6.1
**2APY4a** − e → **2APY4a** ^**-•**^	−18.9	−18.7	0.7	−19.5
**2APY4b** − e → **2APY4b** ^**-•**^	−18.8	−18.6	0.8	−19.4
**4APY1** − e → **4APY1** ^**-•**^	16.7	16.8	0.7	16.1
**4APY2a** − e → **4APY2a** ^**-•**^	−17.2	−17.1	0.5	−17.6
**4APY2b** − e → **4APY2b** ^**-•**^	−17.7	−17.6	0.6	−18.2
**4APY3** − e → **4APY3** ^**-•**^	8.3	8.1	0.1	8.0

^a^ ΔZPE included ^b^ In kcal mol^-1 c^ At 298.15 K

Similar to the proton-transfer interconversions, the entropy terms for both one-electron oxidation (*T*Δ*S*_ox_) and one-electron reduction (*T*Δ*S*_red_) are not very large (≤ 1.3 kcal mol^-1^). This suggests that the electron-transfer processes (electron-loss and electron-gain) are isoentropic in the gas phase for aminopyridines. The relative thermal corrections are also close to zero, and thus, Δ*E*_ox_ ≈ Δ*H*_ox_ ≈ Δ*G*_ox_ and Δ*E*_red_ ≈ Δ*H*_red_ ≈ Δ*G*_red_. If we consider solely the most favored neutral and redox forms of aminopyridines and the favored oxidation and reduction processes (Scheme 3), the following DFT Gibbs energies are found: 183 kcal mol^-1^ for **2APY** − e → **2APY** ^**+•**^, 190 kcal mol^-1^ for **4APY** − e → **4APY** ^**+•**^, 11 kcal mol^-1^ for **2APY** − e → **2APY** ^**-•**^, and 12 kcal mol^-1^ for **4APY** − e → **4APY** ^**-•**^. The oxidation and reduction reactions for **4APY** requires more energy (by 7 and 1 kcal mol^-1^, respectively) than those for **2APY**. Larger difference for the oxidation reaction results from the difference in the tautomeric preferences, **2APY1** ^**+•**^ and **4APY3** ^**+•**^. Interestingly, the literature ionization energy difference for **2APY** and **4APY** is also equal to 7 kcal mol^-1^ (0.3 eV [52]).

### N-aza-effects

**Scheme 4:**
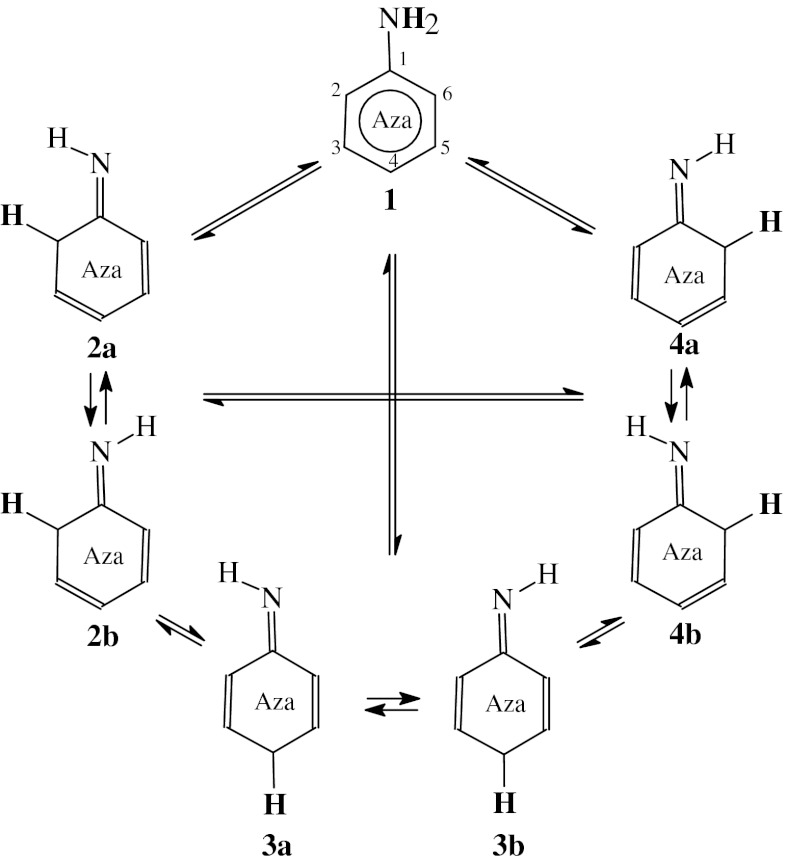
Tautomeric equilibria for azaanilines (labile proton marked in bold)

The amino derivatives of azines studied here (**2APY** and **4APY**) and previously (**4APM**) can be considered as N-aza derivatives of aniline (**AN**). All of them exhibit prototropic tautomerism (Scheme 4). Such kind of treatment of azaanilines gives the possibility to estimate effects of the N-aza group in different position of aniline, 2-N, 4-N and 2,4-N_2_. In this paper, we analyzed the N-aza effects on π-electron delocalization, intramolecular proton-transfer processes (prototropy) and redox reactions for azaanilines. We compared the corresponding parameters (HOMED, p*K*_T_, Δ*G*_ox_, and Δ*G*_red_) calculated at the same level of theory, DFT(B3LYP)/6-311+G(d,p), i.e., those reported in refs [6] and [7] and also those given here in Figs. 4 and 5 and Tables 1, 2, 3, and 4.

Table 5 summarizes the N-aza effects on the HOMED indices estimated for the ring (six bonds) when going from similar isomers of aniline (**AN**) without the N-aza group to those of 2-aminopyridine (**2APY**) and 4-aminopyridine (**4APY**) with one N-aza group in 2- and 4-position, respectively, vis-à-vis the amine group, and to those of 4-aminopyrimidine (**4APM**) with two N-aza groups in 2- and 4-position. The comparison shows that for the amine tautomer **1**, each N-aza group decreases the HOMED indices in higher degree for the anionic than neutral forms (by 0.09 and 0.01 units, respectively), while it increases them for the cationic forms (by ca. 0.02 units). The N-aza effects seem to be additive when going from **AN1** to **2APY1**, **4APY1**, and **4APM1**.

**Table 5:** The N-aza effects (δ) on the HOMED indices estimated for the ring (six bonds) in individual tautomers of azaanilines

Isomer	Charge	δHOMED(2-Ν)	δHOMED(4-Ν)	δHOMED(2,4-Ν_2_)
**1** (NH_2_)	0	−0.004	−0.002	−0.007
	1	0.003	0.021	0.024
	−1	−0.071	−0.011	−0.090
**2a** (NH)	0	-	−0.030	-
	1	-	0.011	-
	−1	-	−0.002	-
**2b** (NH)	0	-	−0.095	-
	1	-	0.011	-
	−1	-	−0.011	-
**3a** (NH)	0	−0.080	-	-
	1	−0.019	-	-
	−1	−0.043	-	-
**3b** (NH)	0	−0.081	-	-
	1	−0.015	-	-
	−1	−0.047	-	-
**3a** (CH)	0	−0.057	-	-
	1	−0.011	-	-
	−1	0.021	-	-
**3b** (CH)	0	−0.061	-	-
	1	0.042	-	-
	−1	−0.028	-	-
**4a** (CH)	0	−0.029	0.019	−0.183
	1	−0.470	0.083	−0.564
	−1	−0.022	0.028	0.000
**4b** (CH)	0	−0.019	−0.011	−0.038
	1	−0.014	−0.028	−0.589
	−1	0.010	0.030	0.067

For the neutral and ionic imine NH tautomers **2** and **3** in **4APM**, the additional N-aza group diminishes the HOMED indices in comparison to **2APY** and **4APY** (by less than 0.1 units). Some exceptions are the cationic isomers **2a** and **2b**, for which the HOMED indices slightly augment (by 0.01 units). The HOMED indices also decease for the neutral and cationic imine CH tautomer **4**. Larger effects take place for the cationic forms (> 0.5 units) than for the neutral forms (< 0.2 units). Some exceptions are the anionic forms, the HOMED indices slightly increase for **4b** (by 0.07 units), however, for **4a** the total effect of the two N-aza groups is equal to zero because one N-aza group decreases and the other N-aza group increases the HOMED index in almost similar degree. In the case of the imine CH tautomer **3**, the N-aza group in 2-position (when going from **AN** to **2APY**) changes the HOMED indices by less than ±0.06 units. However, all these variations of the HOMED indices for tautomers of azaanilines do not change the general trend of π-electron delocalization: the amine and imine NH tautomers are more delocalized than the imine CH ones.

Comparison of the p*K*_T_ values estimated for **AN**, **2APY**, **4APY**, and **4APM** gives some information on the effects of the N-aza group(s) in 2-, 4- and 2,4-positions (Table 6). Generally, the 2-N-aza group increases the p*K*_T_ values for both the NH_2_ → CH and NH_2_ → NH conversions (δ 0–7 p*K*_T_ units) in the neutral and ionic forms of azaanilines. The 4-N-aza group has considerably smaller effect (δ 0 − ±2 p*K*_T_ units). It slightly increases the p*K*_T_ values for the neutral and reduced NH_2_ → CH conversions, and it decreases them for the NH_2_ → NH conversions. The effect of the two N-aza groups seems to be additive only for the neutral forms.

**Table 6:** The N-aza effects (δ) on the p*K*_T_ values estimated for azaanilines

Conversion	Tautomers	δp*K*_T_(2-Ν)	δp*K*_T_(4-Ν)	δp*K*_T_(2,4-Ν_2_)
**1** → **2a**	NH_2_ → CH	-	1.5	-
**1** → **2b**		-	1.1	-
**1** → **3a**		1.7	-	-
**1** → **3b**		4.6	-	-
**1** → **4a**		4.9	1.5	6.2
**1** → **4b**		1.5	1.1	2.9
**1** → **2a**	NH_2_ → NH	-	−0.4	-
**1** → **2b**		-	−0.5	-
**1** → **3a**		1.3	-	-
**1** → **3b**		4.2	-	-
**1** ^**+•**^ → **2a** ^**+•**^	NH_2_ → CH	-	−2.4	-
**1** ^**+•**^ → **2b** ^**+•**^		-	−2.4	-
**1** ^**+•**^ → **3a** ^**+•**^		1.7	-	-
**1** ^**+•**^ → **3b** ^**+•**^		2.5	-	-
**1** ^**+•**^ → **4a** ^**+•**^		7.3	−2.4	0.1
**1** ^**+•**^ → **4b** ^**+•**^		5.3	−2.4	−0.5
**1** ^**+•**^ → **2a** ^**+•**^	NH_2_ → NH	-	−1.9	-
**1** ^**+•**^ → **2b** ^**+•**^		-	−1.7	-
**1** ^**+•**^ → **3a** ^**+•**^		2.7	-	-
**1** ^**+•**^ → **3b** ^**+•**^		6.0	-	-
**1** ^**-•**^ → **2a** ^**-•**^	NH_2_ → CH	-	0.1	-
**1** ^**-•**^ → **2b** ^**-•**^		-	0.1	-
**1** ^**-•**^ → **3a** ^**-•**^		2.4	-	-
**1** ^**-•**^ → **3b** ^**-•**^		5.0	-	-
**1** ^**-•**^ → **4a** ^**-•**^		2.9	0.1	2.3
**1** ^**-•**^ → **4b** ^**-•**^		0.3	0.1	−0.2
**1** ^**-•**^ → **2a** ^**-•**^	NH_2_ → NH	-	−0.5	-
**1** ^**-•**^ → **2b** ^**-•**^		-	−0.4	-
**1** ^**-•**^ → **3a** ^**-•**^		4.4	-	-
**1** ^**-•**^ → **3b** ^**-•**^		7.1	-	-

Independent on the position in the ring, the N-aza group increases the oxidation Gibbs energies (Table 7) and it decreases the reduction Gibbs energies (Table 8). For example, when going from aniline to aminopyridines and 4-aminopyrimidine, the oxidation reaction for the amine tautomer **2APY1**, **4APY1** and **4APM1** requires more energy than that for **AN1** (by 10, 18, and 28 kcal mol^-1^, respectively), whereas lower energy (by 7, 6, and 12 kcal mol^-1^, respectively) is sufficient for the reduction reaction. The effects of the two N-aza groups included in the ring of **4APM1** seem to be additive.

**Table 7:** The N-aza effects (δ) on the Δ*G*_ox_ values estimated for azaanilines

Reaction	Tautomer	δ*G*_ox_(2-Ν)	δ*G*_ox_(4-Ν)	δ*G*_ox_(2,4-Ν_2_)
**1** → **1** ^**+•**^	NH_2_	9.6	17.9	27.9
**2a** → **2a** ^**+•**^	NH	-	16.2	-
**2b** → **2b** ^**+•**^	NH	-	16.6	-
**3a** → **3a** ^**+•**^	NH	12.1	-	-
**3b** → **3b** ^**+•**^	NH	12.3	-	-
**3a** → **3a** ^**+•**^	CH	9.5	-	-
**3b** → **3b** ^**+•**^	CH	6.6	-	-
**4a** → **4a** ^**+•**^	CH	12.9	12.5	19.6
**4b** → **4b** ^**+•**^	CH	13.8	12.7	23.1

**Table 8:** The N-aza effects (δ) on the Δ*G*_red_ values estimated for azaanilines

Reaction	Tautomer	δ*G*_red_(2-Ν)	δ*G*_red_(4-Ν)	δ*G*_red_(2,4-Ν_2_)
**1** → **1** ^**-•**^	NH_2_	−6.7	−5.7	−12.0
**2a** → **2a** ^**-•**^	NH	-	−5.4	-
**2b** → **2b** ^**-•**^	NH	-	−5.2	-
**3a** → **3a** ^**-•**^	NH	−2.0	-	-
**3b** → **3b** ^**-•**^	NH	−2.2	-	-
**3a** → **3a** ^**-•**^	CH	−5.8	-	-
**3b** → **3b** ^**-•**^	CH	−6.1	-	-
**4a** → **4a** ^**-•**^	CH	−9.4	−7.6	−17.1
**4b** → **4b** ^**-•**^	CH	−8.5	−7.2	−16.2

## Conclusions

DFT studies performed for all possible amine and imine tautomers of neutral aminopyridines and its unpaired ions show interesting changes of the tautomeric preferences. Solely for 2-aminopyridine, aromaticity seems to dictate the tautomeric preferences for the neutral and oxidized forms. The amine tautomer **2APY1** ^**+•**^ predominates for the oxidized tautomeric mixture of 2-aminopyridine, similarly as **2APY1** for the neutral molecule. However, one-electron oxidation changes the composition of the tautomeric mixture. The neutral tautomeric mixture consists mainly of **2APY1** (100 %), whereas the oxidized tautomeric mixture contains at least two tautomers: **2APY1** ^**+•**^ (99.9 %) and **2APY2** ^**+•**^ (0.1 %). For comparison, lack of the N-aza group in aniline totally favors **AN1** (100 %) and **AN1** ^**+•**^ (100 %) for the neutral and oxidized tautomeric mixture [6].

In the case of 4-aminopyridine, one-electron oxidation changes the relative stabilities of the unpaired cations containing the labile proton at the N atom. For the neutral tautomeric mixture, **4APY1** is favored (100 %), whereas **4APY3** ^**+•**^ (95.7 %) seems to predominate over **4APY1** ^**+•**^ (4.3 %) for the oxidized tautomeric mixture. Additional N-aza group in 2-position for 4-aminopyrimidine changes the relation between the stabilities of the amine and imine NH tautomers, and **4APM1** ^**+•**^ (88.3 %) predominates over **4APM2** ^**+•**^ (7.3 %) and **4APM3** ^**+•**^ (4.4 %) for the oxidized tautomeric mixtures [7]. The imine CH tautomers may be considered as very rare forms for both neutral and oxidized aminoazines.

The orders of stabilities for the unpaired anion isomers are completely reversed for aminopyridines but similar to those for aniline [6] and 4-aminopyrimidine [7]. The imine CH tautomers are favored for the reduced aminoazines. The tautomeric mixtures of 2- and 4-aminopyridines consist mainly of **2APY4** ^**-•**^ (99.9 %) and **4APY2/4** ^**-•**^ (100 %), respectively. The amounts of **2APY1** ^**-•**^ and **4APY1** ^**-•**^ are not larger than 0.1 %. Similarly, the tautomeric mixtures of aniline and 4-aminopyrimidine consist of **AN2/4** ^**-•**^ (100 %) and **4APM4** ^**-•**^ (99.9 %), respectively. The amounts of **AN1** ^**-•**^ and **4APM1** ^**-•**^ are not larger than 0.1 %.

The importance of the CH tautomers in the tautomeric mixtures of **APY** ^**-•**^ may partially explain the origin of the CH isomers for the anionic states of nucleobases (cytosine and adenine) [54, 55]. On the other hand, the change of the compositions of the tautomeric mixtures for neutral and charged forms of aminoazines should also be taken into account for all processes in which the charged radicals can be formed, e.g., in electrochemical or photochemical processes and during positive or negative ionization in various types of mass spectrometers when the molecule loses or gains one electron [56–58].
